# Protein Tyrosine Phosphatase 1B is Impaired in Skeletal Muscle of Diabetic *Psammomys Obesus*

**DOI:** 10.1080/15604280214275

**Published:** 2002

**Authors:** Yukio Ikeda, Ehud Ziv, Eleazar Shafrir, Luitgard Mosthaf-Seedorf

**Affiliations:** 1 Department of Molecular Signaling Hagedorn Research Institute Gentofte Denmark; 2 Department of Biochemistry and Diabetes Research Unit Hadassah University Hospital Jerusalem 91120 Israel

## Abstract

Protein tyrosine phosphatases (PTPases) have been suggested to modulate the insulin receptor signal transduction pathways.We studied PTPases in *Psammomys obesus*, an animal model of nutritionally induced insulin resistance. No changes in the protein expression level of src homology PTPase 2 (SHP-2) (muscle, liver) or leukocyte antigen receptor (LAR) (liver) were detected. In contrast, the expression level of PTPase 1B (PTP 1B) in the skeletal muscle, but not in liver, was increased by 83% in the diabetic animals, compared with a diabetes-resistant line. However, PTP 1B– specific activity (activity/protein) significantly decreased (50% to 56%) in skeletal muscle of diabetic animals, compared with both the diabetes-resistant line and diabetes-prone animals. In addition, PTP 1B activity was inversely correlated to serum glucose level (r = –.434, *P* < .02). These findings suggest that PTP 1B, though overexpressed, is not involved in the susceptibility to insulin resistance in *Psammomys obesus* and is secondarily attenuated by hyperglycemia or other factors in the diabetic milieu.


*Int. Jnl. Experimental Diab. Res.*, 3:205–212, 2002
Copyright © 2002 Taylor & Francis
1560-4284/02 $12.00 + .00
DOI: 10.1080/15604280290013946

### Protein Tyrosine Phosphatase 1B is Impaired in Skeletal Muscle of Diabetic *Psammomys obesus*

**Yukio Ikeda,[1] Ehud Ziv,[2] Eleazar Shafrir,[2] and Luitgard Mosthaf-Seedorf[1]**

[1]*Department of Molecular Signaling, Hagedorn Research Institute, Gentofte, Denmark*
[2]*Diabetes Research Unit, Hadassah University Hospital, Jerusalem, Israel*

**Protein tyrosine phosphatases (PTPases) have been suggested to modulate the insulin receptor signal transduction pathways. We studied PTPases in *Psammomys obesus*, an animal model of nutritionally induced insulin resistance. No changes in the protein expression level of src homology PTPase 2 (SHP-2) (muscle, liver) or leukocyte antigen receptor (LAR) (liver) were detected. In contrast, the expression level of PTPase 1B (PTP 1B) in the skeletal muscle, but not in liver, was increased by 83% in the diabetic animals, compared with a diabetes-resistant line. However, PTP 1B–specific activity (activity/protein) significantly decreased (50% to 56%) in skeletal muscle of diabetic animals, compared with both the diabetes-resistant line and diabetes-prone animals. In addition, PTP 1B activity was inversely correlated to serum glucose level ($r = -.434$, $P < .02$). These findings suggest that PTP 1B, though overexpressed, is not involved in the susceptibility to insulin resistance in *Psammomys obesus* and is secondarily attenuated by hyperglycemia or other factors in the diabetic milieu.**

**Keywords** *Psammomys obesus*; Protein Tyrosine Phosphatase; PTP 1B; Skeletal Muscle; Sand Rat; Type 2 Diabetes

The desert gerbil *Psammomys obesus* (also known as sand rat) is an animal model of insulin resistance and nutritionally induced diabetes. There is no evidence of hyperglycemia or hyperinsulinemia in animals freshly trapped in their native habitat, where they subsist on a meagre herbivorous diet [1]. The animals remain nondiabetic in captivity when fed a low-energy (LE) diet containing 2.4 cal/g. However, when transferred to a relatively high-energy (HE) diet of 3.0 cal/g, similar to the regular rodent chow, they gradually develop hyperinsulinemia and hyperglycemia. In *Psammomys*, the transition to diabetes have been classified into 4 consecutive stages [2]: stage A, characterized by normoinsulinemia and normoglycemia; stage B, showing hyperinsulinemia without hyperglycemia; stage C, associated with hyperinsulinemia and hyperglycemia; and stage D, representing the terminal stage with hypoinsulinemia and hyperglycemia, which is marked by pancreatic insulin secretion collapse, with apoptosis [3] and dependence on external insulin supply for survival. A certain percentage of the animals do not become diabetic even on a HE diet. Selective breeding resulted in the separation into a diabetes-resistant (DR) line [4], which remains normoglycemic and normoinsulinemic even on a HE diet, and a diabetes-prone (DP) line, which is highly susceptible to diabetes when placed on a HE diet. The hyperglycemia and hyperinsulinemia induced by the HE diet are associated with a decreased tyrosine phosphorylation of insulin receptors (IRs) in liver and muscle and a reduced activity of the IR tyrosine kinase (IR-TK) [5]. Both insulin resistance and impaired tyrosine phosphorylation are reversed upon restriction of food intake [5].

The IR-TK activity is regulated through autophosphorylation of tyrosine residues [6, 7], dephosphorylation by protein tyrosine phosphatases (PTPases) [8, 9], and inhibitory serine/threonine phosphorylation [10, 11]. Thus, the reason for the attenuation of insulin signaling in *Psammomys* could be enhanced PTPase activity and/or increased activity of serine/threonine kinases. The latter possibility was confirmed in a recent study in which we found that protein kinase C$\varepsilon$ (PKC$\varepsilon$) and several other PKC

Received 26 February 2002; accepted 12 May 2002.
The authors thank K. Seedorf for valuable discussions throughout the study and A. Ullrich for the human IR cDNA. The Hagedorn Research Institute is an independent basic research component of Novo Nordisk A/S.
Address correspondence to Eleazar Shafrir, Department of Biochemistry and Diabetes Research Unit, Hadassah University Hospital, Jerusalem 91120, Israel. E-mail: shafrir@md.huji.ac.il isoforms were overexpressed and activated in the skeletal muscle of diabetic *Psammomys* [12]. The overexpression of PKC$\varepsilon$ preceded the onset of hyperinsulinemia and hyperglycemia [12] and could thus be associated with the susceptibility of the animals to insulin resistance.

The aim of our study was to clarify whether alterations in the protein expression level or activity of PTPases could contribute to the development or progression of insulin resistance through dephosphorylation of IR-TK and other components of the signaling pathway. Several PTPases have been found to play a crucial role in regulating insulin signal transduction. Studies in cell models have shown that the leukocyte antigen receptor (LAR) phosphatase, the leukocyte common antigen–related phosphatase (LRP), and PTPase 1B (PTP 1B) can act as negative regulators of IR-TK, whereas src homology PTPase 2 (SHP-2) appears to be a positive mediator-of insulin signaling [9, 13–16]. The observation that PTP 1B–deficient mice showed increased insulin sensitivity and resistance to the development of obesity [17, 18] indicated the possibility of a physiological role for PTP 1B in the negative regulation of insulin action, which had been described previously in a number of in vitro studies [13, 19].

### MATERIALS

Human insulin was provided by Novo Nordisk A/S, Denmark. Protein A sepharose was from Pharmacia Biotech (Sweden). Wheat germ agglutinin (WGA) agarose was from Vector Laboratories (Burlingame, CA). [$\gamma$-$^{32}$P]ATP (3000 Ci/mmol) was from Amersham (UK). Mouse monoclonal antibody against PTP 1B (Ab-1) was purchased from Oncogene Research Products (Cambridge, MA). Rabbit polyclonal SHP-2 antibody and normal mouse immunoglobulin G (IgG) were from Santa Cruz Biotechnology (Santa Cruz, CA). Mouse anti-LAR antibody was from Transduction Laboratories (Lexington, KY). Horseradish peroxidase–conjugated anti-mouse and anti-rabbit IgG were purchased from Amersham. The cDNA for the type A IR [20] was cloned into a cytomegalovirus promotor-enhancer–driven expression vector.

### Experimental Animals and Dietary Treatment

Male and female *Psammomys obesus* from the DP and DR lines were bred at the Hebrew University Hadassah Medical School Animal Farm. The animals were weaned at 3 weeks of age to a LE or HE diet (2.38 and 2.93 cal/g, respectively) manufactured by Koffolk, Petach Tikva, Israel. *Psammomys* were housed in individual polypropylene cages, water and food being supplied ad libitum. All experimental procedures performed in this study were authorized by the Institutional Animal Care Committee.

### METHODS

### Tissue and Blood Collection

The animals were killed by decapitation in the fed state in the morning. Blood was collected for the determination of serum glucose and triglycerides by enzymatic methods (Roche, Basel, Switzerland) and serum insulin by radioimmunoassay, utilizing anti-human antibodies (Medgenix, Brussels, Belgium). The liver and gastrocnemius muscle from both legs were excised and immediately frozen in liquid nitrogen, then stored at −80°C.

### Preparation of Lysates

Muscle and liver tissues were extracted in ice-cold homogenization buffer I (final concentrations: 20 mM Tris-Acetate, pH 7.0, 0.27 M sucrose, 1 mM EDTA, 1 mM EGTA, 1 mM 4(aminoethyl)benzenesulphonyl fluoride (AEBSF), 0.5% Triton X-100, 1 mM benzamidine, 1 mM dithiothreitol (DTT), and 4 $\mu$g/mL leupeptin) using a Polytron. After 20 minutes at 4°C, the samples were centrifuged at 20,000 × *g* for 60 minutes to remove insoluble material and the clarified extracts were stored at −80°C.

### Subcellular Fractionation

Approximately 200 mg of powdered muscle was homogenized in 2 mL of homogenization buffer II (20 mM Tris-Acetate, pH 7.0, 0.27 M sucrose, 1 mM EDTA, 1 mM EGTA, 1 mM AEBSF, 1 mM benzamidine, 1 mM DTT, and 4 $\mu$g/mL leupeptin) using a Polytron. Samples were maintained on ice during subsequent steps and all centrifugations were performed at 4°C. The crude homogenate was centrifuged at 700 × *g* for 10 minutes, and the resultant supernatant was centrifuged at 183,000 × *g* for 60 minutes. The supernatant was obtained and designated as the cytosolic fraction. The pellet was solubilized in buffer I for 30 minutes and was centrifuged at 183,000 × *g* for 60 minutes to obtain solubilized membrane fraction. The protein concentration was determined using the method of Bradford (BioRad protein assay, BioRad Laboratories, Richmond, CA), and the tissue fractions were diluted to 1 mg/mI and then stored at −80°C.

### Western Blotting, Enhanced Chemical Luminescence, and Quantification of the Bands

Equal amounts of tissue homogenates (40 $\mu$g/sample) were dissolved in 2× Laemmli buffer and subject to sodium dodecyl sulfate–polyacrylamide gel electrophoresis (SDS-PAGE). The proteins were transferred to nitrocellulose membranes (BA85, Schleicher & Schüll, Dassel, Germany). Immunoreactive proteins were made visible using horseradish peroxidase–coupled secondary antibodies and enhanced chemiluminescence (ECL) reagents according to the manufacturer's (Amersham) instructions. The specific bands were quantified using ImageQuant software (Molecular Dynamics, Sunnyvale, CA).

### Cell Culture, Transfection, and Transient Expression of Human Insulin Receptors

Human embryonic kidney fibroblasts (HEK293; ATTC CRL 1573, ATCC, Rockville, MD) were cultured in Dulbecco's modified Eagles medium supplemented with 10% (*v*/*v*) fetal bovine serum (Life Technologies, Eggestein, Germany), 10 mg/mL streptomycin, and 100 U/mL penicillin at 37°C in a 5% $CO_2$-enriched, humidified atmosphere. The cells were transiently transfected with IR expression plasmid using $CaCl_2$ as previously described by Chen and Okayama [21] and Gorman and coworkers [22]. Twenty-four hours after transfection, the cells were starved overnight in medium containing 5 mM glucose and 0.5% fetal bovine serum.

### Cell Lysis and Preparation of Phosphorylated Insulin Receptors

HEK293 cells overexpressing IRs were homogenized in lysis buffer I containing 20 mM Hepes buffer pH 7.5, 150 mM NaCl, 10% glycerol, 1.5 mM $MgCl_2$, 4 mM EGTA, 1 mM EDTA, 1% Triton X-100, 1 mM AEBSF, 30 mM Na 2P2O4, 100 mM Na 3VO4, 10 mM NaF, 5 ug/ml leupeptin and 10 mM benzamidine. Cellular debris was removed by centrifugation at 15,000 × *g* for 10 minutes at 4°C. IRs were purified by wheat germ agglutinin chromatography of the cell lysates. Aliquots of 120 $\mu$g protein of partially purified IRs were autophosphorylated in a reaction containing 1 $\mu$M insulin, 5 mM $MnCl_2$, 100 $\mu$M ATP, 180 $\mu$Ci/tube of [$\gamma$-$^{32}$P]ATP, and 0.1% Triton X-100 in 50 mM Hepes buffer, pH 7.6, at 4°C for 120 minutes. Unincorporated [$\gamma$-$^{32}$P]ATP was removed using rapid gel on a small Sephadex G-25 column equilibrated in reaction buffer containing 50 mM Hepes, pH 7.6, 1 mM DTT, and 2 mM EDTA.

### Measurements of Tissue PTPase Activity

Aliquots of the labeled receptors (2 $\mu$g/sample) were incubated with 40 $\mu$g protein of the samples in the reaction buffer at 30°C for 20 minutes. The reactions were terminated by adding 5× Laemmli buffer and boiling the samples at 100°C for 2 minutes. The samples were subjected to SDS-PAGE. Dephosphorylation of the 95-kDa $\beta$-subunit of the IR was analyzed using PhosphorImager (Molecular Dynamics).

### Measurement of PTP 1B Activity

PTP 1B activity was determined by immunoprecipitation using the antibody against PTP 1B catalytic domain. Aliquots of tissue homogenates (400 $\mu$g) were incubated with 2 $\mu$g of anti–PTP 1B antibody or normal mouse IgG (as control) at 4°C for 120 minutes. The complexes were precipitated by incubation with protein A sepharose for 120 minutes. After centrifugation at 15,000 × *g* for 2 minutes, PTPase activity in the supernatants was measured as described above. Figure 1 shows the efficiency for immunoprecipitation with this antibody, which resulted in

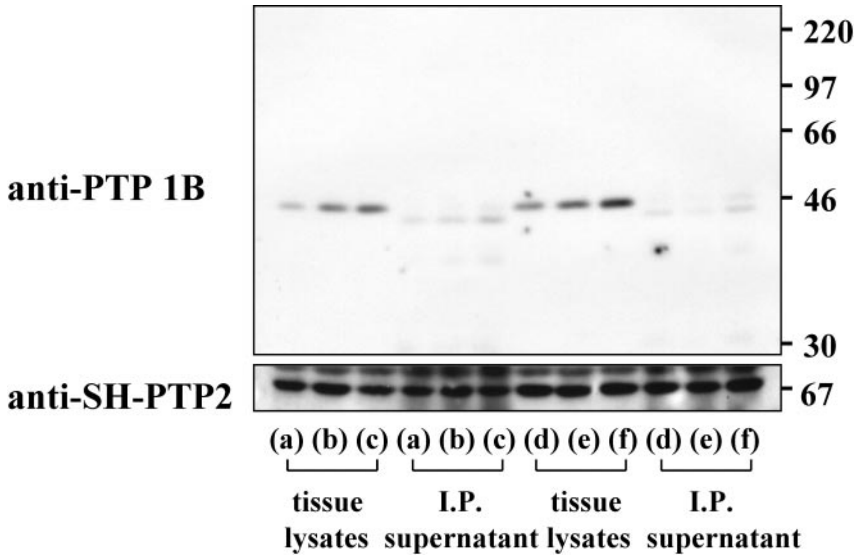

**FIGURE 1**
Specificity of mouse monoclonal antibody toward PTP 1B and its efficiency for immunoprecipitation. Muscle homogenates were incubated with anti–PTP 1B antibody, then precipitated with protein A sepharose. The supernatants (40 $\mu$g protein) were subjected to 10% SDS-PAGE and probed with anti–PTP 1B antibody. Equal amounts of total homogenates were also subjected to the gel to investigate the efficiency for immunoprecipitation of this antibody. The membrane was reprobed with anti–SHP-2 antibody as an endogenous control. (a), (d) are from the diabetes-resistant (DR) line; (b), (e) are from the diabetes-prone (DP) line, stage A; and (c), (f) are from the diabetes-prone (DP) line, stage C. I.P.: immunoprecipitation.

**TABLE 1**
Animal characteristics

| Animal/stage (n) | Weight (g) | Maintenance diet | Serum glucose (mmol/L) | Insulin (pmol/L) |
|---|---|---|---|---|
| DR (9) | $207 \pm 8^{***}$ | HE | $5.0 \pm 0.3^{**}$ | $947 \pm 158^{*}$ |
| DP/A (11) | $151 \pm 5$ | LE | $4.1 \pm 0.2$ | $581 \pm 65$ |
| DP/C (10) | $201 \pm 7^{***}$ | HE | $14.7 \pm 1.1^{***,\ddagger}$ | $2497 \pm 352^{***,\ddagger}$ |

*Note*. The table summarizes weight and serum glucose and insulin levels in the 3 different groups of *Psammomys obesus* used in this study. The values were obtained in nonfasting animals. DR: diabetes resistant; DP/A: diabetes-prone stage A (normoinsulinemic, normoglycemic); DP/C: diabetes-prone stage C (hyperinsulinemic, hyperglycemic). "n" indicates the number of individuals within the group. Values are the mean ± SEM. $^{*}P < .05$, $^{**}P < .01$, $^{***}P < .001$ versus DP/A; $^{\ddagger}P < .001$ versus DR.

depletion of 90.7% to 93.7% of PTP 1B expression compared with that in total muscle homogenates. The difference in PTPase activity between immunodepleted supernatant with normal mouse IgG and that with anti–PTP 1B was calculated and defined as PTP 1B activity.

### Statistical Analysis

All values are presented as the mean ± SEM. Group comparisons were made by unpaired Student's *t* test. Correlations were assessed by Pearson's least squares method. *P* values less than .05 were considered significant.

### RESULTS

### Animal Characteristics

The general characteristics of animals used in this study are summarized in Table 1. DP animals on HE diet (3.0 cal/g) showed hyperinsulinemia and hyperglycemia. However, DP animals on LE diet (2.4 cal/g) had relatively lower body weights and glucose and insulin levels than DR animals on HE diet. Well-defined animals groups ($n = 9$–11 per group) were taken for the PTPase study.

### PTP 1B Overexpression in the Gastrocnemius Muscle of Diabetic *Psammomys obesus*

We first aimed to determine the protein expression level of specific PTPase enzymes (PTP 1B, SHP-2, and LAR) in skeletal muscle tissues. Western blots of muscle lysates were probed with specific antibodies for these PTPases. An example of such a Western blot for PTP 1B is shown for a subset of samples from each group in Figure 1 (tissue lysates a to f). PTP 1B was detected as a single band at ~50 kDa. No bands corresponding to LAR were detected in skeletal muscle, although clear

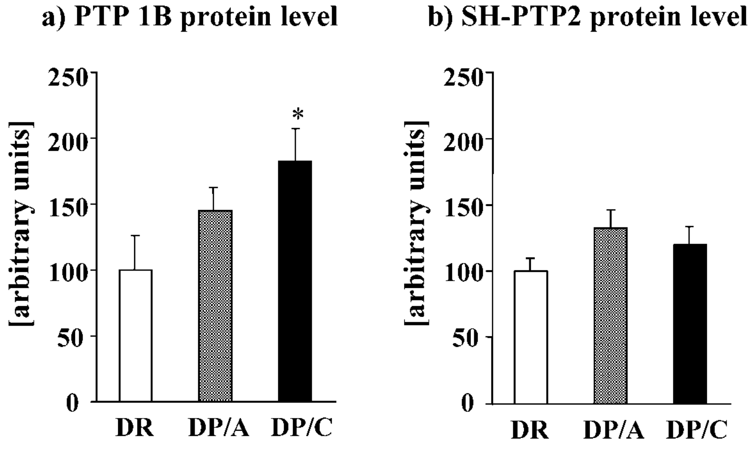

**FIGURE 2**
Expression of PTP 1B and SHP-2 in the gastrocnemius muscle. Muscle homogenates (40 $\mu$g) were subjected to SDS-PAGE and Western blotting, then probed with SHP-2– or PTP 1B–specific antibodies. The respective bands were quantitated using ImageQuant software. The mean value of DR animals was set to 100 and all other values were calculated accordingly. Expression levels given represent the mean ± SEM of 9 to 11 samples. *P* values were determined using Student's *t* test. DR: diabetes-resistant line; DP/A: diabetes-prone line, stage A (normoinsulinemic, normoglycemic); DP/C: diabetes-prone line, stage C (hyperinsulinemic, hyperglycemic). $^{*}P < .05$ versus DR.

single bands at ∼150 kDa were detected in liver homogenates. Figure 2 summarizes the quantification and statistical analysis of the specific bands from all samples analyzed. Expression levels of PTP 1B were significantly increased in the diabetic animals (DP/C) compared with the DR line (82.9% increase), and the increase was also observed in the prediabetic animals (DP/A), but not significantly so. There was no significant difference in SHP-2 expression between healthy and diabetic animals (data not shown).

### Total PTPase Activity

Because PTP 1B was overexpressed in skeletal muscle of diabetic *Psammomys*, we determined the PTPase activity in each group. The enzyme activity, measured using IR as a substrate, was surprisingly decreased in diabetic animals (DP/C) compared with DR (27.5% decrease) or DP/A animals (22.5% decrease) (Figure 3*a*). We also measured the PTPase activity in subcellular fractions and found that this decrease was mainly due to the decrease in the particulate fraction (DR = 100.0 ± 12.8; DP/A = 93.7 ± 5.9; DP/C = 51.9 ± 13.6 [arbitrary units], $P < .05$).

We further investigated whether the decrease in the overall PTPase activity in diabetic animals was associated with a change in PTP 1B activity. PTP 1B activity was calculated as total PTPase activity minus PTPase activity after depletion with a PTP 1B–specific antibody. In addition to the decrease in total PTPase activity, we detected also a decrease in PTP 1B activity in muscle homogenates of diabetic animals (Figure 3*b*). PTP 1B activity in the particulate fraction was decreased to 60% in diabetic animals when compared with DR or DP/A animals (DR = 53.5 ± 6.7; DP/A = 53.5 ± 5.3; DP/C = 32.3 ± 5.6 [arbitrary units], $P < .05$). However the decrease was not significant when compared with DR animals. The ratio between protein expression and activity, which could reflect the enzyme activity per molecule, was markedly decreased in diabetic animals (0.23 ± 0.05) compared with both DR and DP/A animals (0.52 ± 0.12 and 0.45 ± 0.09, respectively) (Figure 3*c*), suggesting attenuation of this molecule by the diabetic milieu. There was an inverse correlation between PTP 1B activity in skeletal muscle and serum glucose concentration ($r = -.434$, $P < .02$). There was also an inverse relationship between total PTPase activity in skeletal muscle and serum glucose concentration ($r = -.625$, $P < .001$) (Figure 4).

### Total Liver PTPase Activity

We also examined PTPase expression and activity in liver homogenates ($n = 9$–10 per group). No significant differences in the expression levels of LAR, SHP-2, or PTP 1B in the 3 groups of animals were detected (data not shown). In these samples, total PTPase activity in diabetic animals was significantly

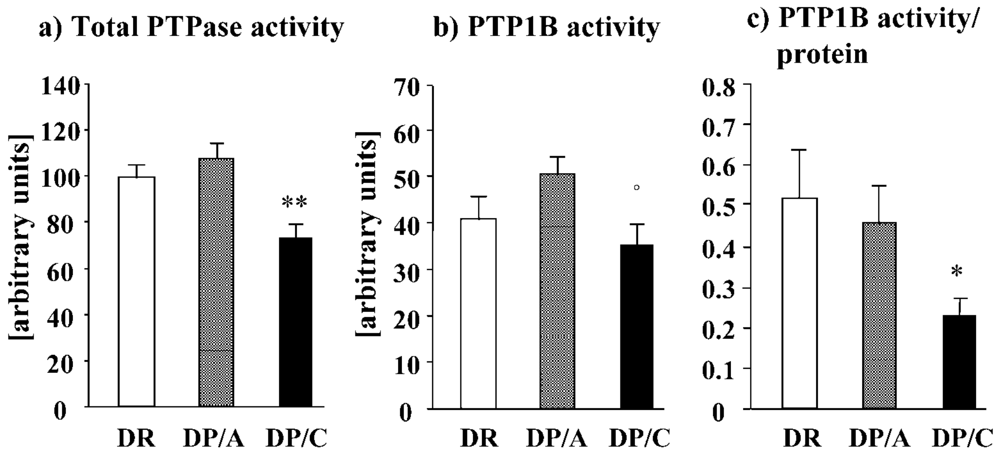

**FIGURE 3**

PTPase activity in the gastrocnemius muscle. (*a*) Total PTPase activity in muscle homogenates was measured as described in Methods, using autophosphorylated insulin receptor as the substrate. Dephosphorylation of the 95-kDa $\beta$-subunit of the insulin receptor was analyzed by PhosphorImager (Molecular Dynamics). (*b*) Muscle homogenates were precipitated with anti–PTP 1B antibody or normal mouse IgG (control) and protein A sepharose. PTPase activities in the supernatants were measured and the difference in the enzyme activity between 2 samples was calculated and designated as the PTP 1B activity. (*c*) PTP 1B activities in muscle homogenates were divided by its expression in the individual samples to reflect the enzyme activity per molecule. The mean value of total PTPase activity in DR animals was set to 100 and all other values were calculated accordingly. Data represent the mean ± SEM of 9 to 11 samples for (*a*) and 8 to 10 samples for (*b*), (*c*). *P* values were determined using Student's *t* test. DR: diabetes-resistant line; DP/A: diabetes-prone line, stage A (normoinsulinemic, normoglycemic); and DP/C: diabetes-prone line, stage C (hyperinsulinemic, hyperglycemic). $^{*}P < .05$, $^{**}P < .01$ versus DR; $^{\dagger}P < .05$, $^{\ddagger}P < .01$ versus DP/A.

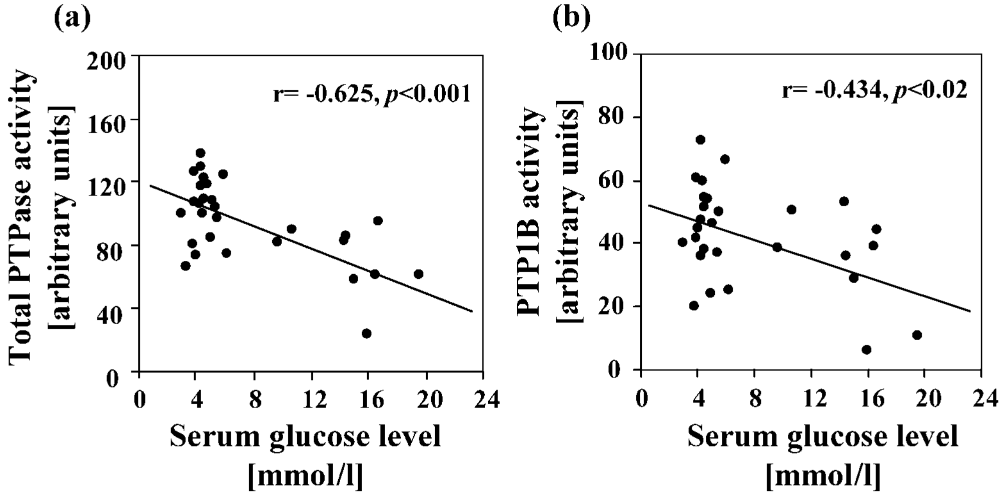

**FIGURE 4**

Correlation between PTPase activity in skeletal muscle and serum glucose concentrations. The activities of total PTPase and PTP 1B were determined as described in Methods and the correlations in the individual *Psammomys* samples ($n = 29$) are plotted. Correlation coefficients ($r$) and $P$ values are indicated.

lower compared with that in DR and DP/A animals (DR = 100.0 ± 3.9; DP/A = 106.5 ± 4.1; DP/C = 82.5 ± 1.9 [arbitrary units], $P < .001$). There was no significant difference in PTP 1B activity (DR = 38.0 ± 1.7; DP/A = 42.5 ± 1.6; DP/C = 37.8 ± 1.9 [arbitrary units]).

### DISCUSSION

PTP 1B has been associated with insulin resistance in different cell models [23–26]. Some studies have reported the influence of hyperinsulinemia or hyperglycemia on PTP 1B content or PTPase activity in vitro [27, 28]. Kenner and colleagues [27] showed that insulin increased particulate PTPase activity and PTP 1B mRNA and protein in rat L6 skeletal muscle cells. Ide and coworkers [28] reported that D-glucose increased cytosolic PTPase activity and PTP 1B content. However, the PTP 1B expression level or PTPase activity in insulin-sensitive tissues of insulin-resistant animals or humans is not uniform [29–37] and the significance is unknown. The reported variation of PTPase activity may be caused by differences in species, tissue, period, or level of hyperglycemia. The difference in substrate used for the PTPase assay may also result in such variation. For example, Ahmad and Goldstein [29] demonstrated that the particulate PTPase activity toward both myelin basic protein and lysozyme was increased in hindlimb skeletal muscle of insulin-resistant obese (*fa*/*fa*) and diabetic (ZDF/Drt-*fa*/*fa*) Zucker rats. In contrast, Worm and colleagues [35] demonstrated that cytosolic PTPase activity toward immobilized IRs was decreased in the soleus muscle of *fa*/*fa* Zucker rats, whereas the particulate PTPase activity did not change.

We investigated the protein expression level and activity of PTP 1B in *Psammomys obesus*, which has the advantage of being extremely well characterized with respect to the development of insulin resistance and diabetes. Our findings suggest that PTP 1B is overexpressed in the skeletal muscle of diabetic *Psammomys obesus* (DP/C), using animals from the diabetes-resistant line (DR) as a basal reference. This appears to be a specific change because expression levels of SHP-2, a positive regulator of insulin signaling, were not increased. We measured PTPase activity using whole IR as the substrate. Impairment of the IR-TK activity in stages B and C has been shown before [5], and we, therefore, suggested that this might be causally related to alterations in PTPase expression or activity. In contrast to our expectations, we found that PTPase activity toward autophosphorylated IR was significantly decreased in diabetic animals compared with not only the DR animals but also the diabetes-prone DP/A animals. To assess PTP 1B activity, we immunoprecipitated using anti-PTP 1B–specific antibody and measured PTPase activity in the depleted supernatant. Because this antibody efficiently precipitates PTP 1B from tissue homogenate, we consider this method suitable for evaluating PTP 1B activity in these animals. The specific activity of PTP 1B (activity/protein) was significantly decreased in DP/C compared with DR and DP/A animals. The total activity of PTP 1B was significantly decreased in DP/C compared with DR and DP/A animals (normoglycemic, normoinsulinemic *Psammomys* from the same line). In addition, total PTP 1B activity was decreased in the particulate fraction to 60% in DP/C when compared with DR and DP/A animals. These findings suggest that PTP 1B is impaired in skeletal muscle of diabetic animals and, therefore, most likely rules out a role for this PTPase in induction of insulin resistance through increased dephosphorylation of the IR.

Worm and colleagues [35] reported that skeletal muscle PTPase activity was decreased in diabetic (*fa*/*fa*) Zucker rats compared with their lean littermates (*Fa*/−). The reduction was completely prevented by metformin treatment, which decreased plasma glucose and insulin levels. This appears to concur with the present observation that the reduction in PTPase activity in DP animals was fully prevented by dietary restriction, which kept them normoinsulinemic and normoglycemic. These findings suggest that the difference in PTP 1B activity in skeletal muscle between the diabetic *Psammomys* and the controls is not genetic, but a secondary change to diabetes mellitus, and that the overexpression of PTP 1B probably results from a compensatory mechanism.

The inverse correlation between serum levels and PTP 1B activity (Figure 4, showing lower PTPase activity at higher glucose levels), may be interpreted to be the result of a deleterious effect of the diabetic milieu on PTP 1B activity. Glycation and/or oxidative inactivation [38] of the enzyme are probably detrimental to its activity. We therefore do not believe that the high glucose levels are attenuating the insulin action through PTPase activity. Further investigation is required to clarify this.

Because the liver plays an important role in regulating blood glucose levels through gluconeogenesis, we also examined the expression and activity of PTPase in this tissue. However, there was no difference in expression levels of PTP 1B, SHP-2, or LAR among the three groups. Total PTPase activity in the liver was decreased in diabetic animals, but this decrease could not be explained by reduced PTP 1B enzyme activity. In our companion article in this issue, Meyerovitch and colleagues [39] reported that hepatic cytosolic PTPase LAR significantly increased after overnight fasting in nondiabetic *Psammomys*, but not in diabetic animals, perhaps due to a regulatory defect. Therefore, a regulatory PTPase defect in the diabetic state may have a more important impact than an alteration in basal activity.

In conclusion, we have demonstrated that diabetic *Psammomys obesus* (DP/C) exhibited an impaired PTP 1B (protein overexpression with decreased activity) in skeletal muscle. This was not detected in DP/A animals from the same line, which were normoinsulinemic and normoglycemic on a LE diet. In general, PTP 1B does not appear to be causally related to the difference in susceptibility to diabetes between the DR and DP lines, and its activity does not impair IR-TK activity in *Psammomys obesus*. The up-regulated expression of skeletal muscle PTP 1B may probably be a result of compensation for the attenuated activity in diabetes. Further studies are required to uncover the mechanism of PTP 1B attenuation in diabetes.

### REFERENCES

[1] Ziv, E., and Shafrir, E. (1995) *Psammomys obesus* (sand rat): Nutritionally induced NIDDM-like syndrome on a thrifty gene background. In: *Lessons from Animal Diabetes*, edited by Shafir, E., vol. 5, pp 285–300. London, Smith-Gordon.

[2] Kalderon, B., Gutman, A., Shafrir, E., and Adler, J. H. (1986) Characterization of stages in the development of obesity-syndrome in sand rat (*Psammomys obesus*). *Diabetes,* **35,** 717–724.

[3] Bar-On, H., Ben Sasson, R., Ziv, E., and Shafrir, E. (1999) Irreversibility of nutritionally induced NIDDM in *Psammomys obesus* is related to beta-cell apoptosis. *Pancreas,* **18,** 259–265.

[4] Kalman, R., Adler, J. H., Lazarovici, G., Bar-On, H., and Ziv, E. (1993) The efficiency of sand rat metabolism in responsible for development of obesity and diabetes. *J. Basic Clin. Physiol. Pharmacol.,* **4,** 57–68.

[5] Kanety, H., Moshe, S., Shafrir, E., Lunenfeld, B., and Karasik, A. (1994) Hyperinsulinemia induces a reversible impairment in insulin receptor function leading to diabetes in the sand rat model of non-insulin dependent diabetes. *Proc. Natl. Acad. Sci. U. S. A.,* **91,** 1853–1857.

[6] Kasuga, M., Karlsson, F. A., and Kahn, C. R. (1982) Insulin stimulates the phosphorylation of the 95,000-dalton subunit of its own receptor. *Science,* **215,** 185–187.

[7] Rosen, O. M. (1987) After insulin binds. *Science,* **237,** 1452–1458.

[8] White, M. F., and Kahn, C. R. (1994) The insulin signaling system. *J. Biol. Chem.,* **269,** 1–4.

[9] Goldstein, B. J. (1993) Regulation of insulin receptor signaling by protein-tyrosine dephosphorylation. *Receptor,* **3,** 1–15.

[10] Berti, L., Mosthaf, L., Kroder, G., Mushack, J., Seffer, E., Seedorf, K., and Häring, H. U. (1994) Glucose induced translocation of protein kinase C isoforms in rat-1 fibroblasts is paralleled by inhibition of the insulin receptor tyrosine kinase. *J. Biol. Chem.,* **269,** 3381–3386.

[11] Bossenmaier, B., Mosthaf, L., Mischak, H., Ullrich, A., and Häring, H. U. (1997) Protein kinase C isoforms $\beta 1$ and $\beta 2$ inhibit the tyrosine kinase activity of the insulin receptor. *Diabetologia,* **40,** 863–866.

[12] Ikeda, Y., Olsen, G. S., Ziv, E., Hansen, L. L., Busch, A. K., Hansen, B. F., Shafrir, E., and Mosthaf-Seedorf, L. (2001) Cellular mechanism of nutritionally induced insulin resistance in *Psammomys obesus*: Overexpression of protein kinase C epsilon in skeletal muscle precedes the onset of hyperinsulinemia and hyperglycemia. *Diabetes,* **50,** 584–592.

[13] Goldstein, B. J., Ahmad, F., Ding, W., Li, P.-M., and Zhang, W.-R. (1998) Regulation of the insulin signalling pathway by cellular protein-tyrosine phosphatases. *Mol. Cell. Biochem.,* **182,** 91–99.

[14] Maegawa, H., Hasegawa, M., Sugai, S., Obata, T., Ugi, S., Morino, K., Egawa, K., Fujita, T., Sakamoto, T., Nishio, Y., Kojima, H., Haneda, M., Yasuda, H., Kikkawa, R., and Kashiwagi, A. (1999) Expression of a dominant negative SHP-2 in transgenic mice induces insulin resistance. *J. Biol. Chem.,* **274,** 30236–30243.

[15] Kennedy, B. P. (1999) Role of protein tyrosine phosphatase-1B in diabetes and obesity. *Biomed. Pharmacother.,* **53,** 466–470.

[16] Kennedy, B. P., and Romachandran, C. (2000) Protein tyrosine phosphatase-1B in diabetes. *Biochem. Pharmacol.,* **60,** 877–883.

[17] Elchebly, M., Payette, P., Michaliszyn, E., Cromlish, W., Collins, S., Loy, A. L., Normandin, D., Cheng, A., Himms-Hagen, J., Chan, C.-C., Ramachandran, C., Gresser, M. J., Tremblay, M. L., and Kennedy, B. P. (1999) Increased insulin sensitivity and obesity resistance in mice lacking the protein tyrosine phosphatase-1B gene. *Science,* **283,** 1544–1548.

[18] Klaman, L. D., Boss, O., Peroni, O. D., Kim, J. K., Martino, J. L., Zabolotny, J. M., Moghal, N., Lubkin, M., Kim, Y.-B., Sharpe, A. H., Stricker-Krongrad, A., Shulman, G. I., Neel, B. G., and Kahn, B. B. (2000) Increased energy expenditure, decreased adiposity, and tissue-specific insulin sensitivity in protein-tyrosine phosphatase 1B-deficient mice. *Mol. Cell. Biol.,* **20,** 5479–5489.

[19] Goldstein, B. J., Bittner-Kowalczyk, A., White, M. F., and Harbeck, M. (2000) Tyrosine dephosphorylation and deactivation of insulin receptor substrate-1 by protein-tyrosine phosphatase 1B. *J. Biol. Chem.,* **275,** 4283–4289.

[20] Ebina, Y., Ellis, L., Jarnagin, K., Edery, M., Graf, L., Clauser, E., Ou, J. H., Masiarz, F., Kan, Y. W., Goldfine, I. D., Roth, R., and Rutter, W. (1985) The human insulin receptor cDNA: The structural basis for hormone-activated transmembrane signalling. *Cell,* **46,** 747–758.

[21] Chen, C., and Okayama, H. (1987) High-efficiency transformation of mammalian cells by plasmid DNA. *Mol. Cell. Biol.,* **7,** 2745–2752.

[22] Gorman, C. M., Gies, D., McCray, G., and Huang, M. (1989) The human cytomegalovirus major immediate early promoter can be trans-activated by adenovirus early proteins. *Virology,* **171,** 377–385.

[23] Cicirelli, M. F., Tonks, N. K., Diltz, C. D., Weiel, J. E., Fischer, E. H., and Krebs, E. G. (1990) Microinjection of a protein-tyrosine phosphatase inhibits inhibits insulin action in *Xenopus* oocytes. *Proc. Natl. Acad. Sci. U. S. A.,* **87,** 5514–5518.

[24] Ahmad, F., Li, P. M., Meyerovitch, J., and Goldstein, B. J. (1995) Osmotic loading of neutralizing antibodies demonstrates a role for protein-tyrosine phosphatase 1B in negative regulation of the insulin action pathway. *J. Biol. Chem.,* **270,** 20503–20508.

[25] Kenner, K. A., Anyanwu, E., Olefsky, J. M., and Kusari, J. (1996) Protein-tyrosine phosphatase 1B is a negative regulator of insulin- and insulin-like growth factor-I-stimulated signaling. *J. Biol. Chem.,* **271,** 19810–19816.

[26] Chen, H., Wertheimer, S. J., Lin, C. H., Katz, S. L., Amrein, K. E., Bum, P., and Quon, M. J. (1997) Protein-tyrosine phosphatases PTP-1B and Syp are modulators of insulin-stimulated translocation of GLUT4 in transfected rat adipose cells. *J. Biol. Chem.,* **272,** 8026–8031.

[27] Kenner, K. A., Hill, D. E., Olefsky, J. M., and Kusari, J. (1993) Regulation of protein tyrosine phosphatases by insulin and insulin-like growth factor I. *J. Biol. Chem.,* **268,** 25455–25462.

[28] Ide, R., Maegawa, H., Kikkawa, R., Shigeta, Y., and Kashiwagi, A. (1994) High glucose condition activates protein tyrosine phosphatases and deactivates insulin receptor function in insulin-sensitive Rat 1 fibroblasts. *Biochem. Biophys. Res. Commun.,* **201,** 71–77.

[29] Ahmad, F., and Goldstein, B. J. (1995) Increased abundance of specific skeletal muscle protein-tyrosine phosphatases in a genetic model of insulin-resistant obesity and diabetes mellitus. *Metabolism,* **44,** 1175–1184.

[30] Cheung, A., Kusari, J., Jansen, D., Bandyopadhyay, D., Kusari, A., and Bryer-Ash, M. (1999) Marked impairment of protein tyrosine phoaphatase 1B activity in adipose tissue of obese subjects with and without type 2 diabetes mellitus. *J. Lab. Clin. Med.,* **134,** 115–123.

[31] Kusari, J., Kenner, K. A., Suh, K. I., Hill, D. E., and Henry, R. R. (1994) Skeletal muscle protein tyrosine phosphatase activity and tyrosine phosphatase 1B protein content are associated with insulin action and resistance. *J. Clin. Invest.,* **93,** 1156–1162.

[32] Ahmad, F., Azevedo, J. L., Cortright, R., Dohm, G. L., and Goldstein, B. J. (1997) Alteration in skeletal muscle protein-tyrosine phosphatase activity and expression in insulin-resistant human obesity and diabetes. *J. Clin. Invest.,* **100,** 449–458.

[33] Begum, N., Sussman, K. E., and Draznin, B. (1991) Differential effect of diabetes on adipocyte and liver phosphotyrosine and phosphoserine phosphatase activities. *Diabetes,* **40,** 1620–1629.

[34] Olichon-Berthe, C., Haugel-De Mouzon, S., Peraldi, P., Van Obberghen, E., and Le Marchand-Brustel, Y. (1994) Insulin receptor dephosphorylation by phosphotyrosine phosphatases obtained from insulin-resistant obese mice. *Diabetologia,* **37,** 56–60.

[35] Worm, D., Handberg, A., Hoppe, E., Vinten, J., and Beck-Nielsen, H. (1996) Decreased skeletal muscle phosphotyrosine phosphatase (PTPase) activity towards insulin receptors in insulin-resistant Zucker rats measured by delayed europium fluorescence. *Diabetologia,* **39,** 142–148.

[36] Meyerovitch, J., Rothenberg, P., Shechter, Y., Bonner-Weir, S., and Kahn, C. R. (1991) Vanadate normalizes hyperglycemia in two mouse models of non-insulin-dependent diabetes mellitus. *J. Clin. Invest.,* **87,** 1286–1294.

[37] Worm, D., Vinten, J., Staehr, P., Henriksen, J. E., Handberg, A., and Beck-Nielsen, H. (1996) Altered basal and insulin-stimulated phosphotyrosine phosphatase (PTPase) activity in skeletal muscle from NIDDM patients compared with control subjects. *Diabetologia,* **39,** 1208–1214.

[38] Mahadev, K., Zilbering, A., Zhu, L., and Goldstein, B. J. (2001) Insulin-stimulated hydrogen peroxide reversibly inhibits protein-tyrosine phosphatase 1B in vivo and enhances the early insulin action cascade. *J. Biol. Chem.,* **276,** 21938–21942.

[39] Meyerovitch, J., Balta, Y., Ziv, E., Sack, J., and Shafrir, E. (2002) Protein tyrosine phosphatase activity in insulin-resistant rodent *Psammomys obesus*. *Int. J. Exp. Diabetes Res.,* **3,** 199–204.

